# Use and overuse of positron emission tomography for patients with metastatic colorectal cancer

**DOI:** 10.1093/oncolo/oyag336

**Published:** 2026-08-21

**Authors:** Daniel A Goldstein, Roi Tschernichovsky, Talish Razi, Ronen Arbel, Doron Netzer

**Affiliations:** Center for Cancer Economics, Davidoff Cancer Center, Rabin Medical Center, Petah Tikva, 4941492, Israel; Gray Faculty of Medical and Health Sciences, Tel Aviv University, Tel Aviv, 6997801, Israel; Davidoff Cancer Center, Rabin Medical Center, Petah Tikva, 4941492, Israel; Department of Molecular Cell Biology, Weizmann Institute of Science, Rehovot, 7632701, Israel; Clalit Health Service, Tel Aviv, 6468211, Israel; Clalit Health Service, Tel Aviv, 6468211, Israel; Sapir College, Sderot, 7956000, Israel; Clalit Health Service, Tel Aviv, 6468211, Israel

**Keywords:** colorectal cancer, healthcare resource allocation, PET/CT, retrospective study

## Abstract

In all healthcare systems, and particularly those that are resource limited, using diagnostic techniques efficiently is essential. According to consensus guidelines, standard computed tomography (CT) imaging without positron emission tomography (PET) is usually sufficient for the management of metastatic colorectal cancer (mCRC). This retrospective national cohort study evaluated the use of PET in mCRC management among members of Clalit Health Services (CHS, the largest Israeli health maintenance organization covering >50% of the Israeli population), utilizing the CHS central database. The cohort included CHS members diagnosed with mCRC between 2017 and 2022 (*N* = 998). The median number of PETs performed per patient was 5. Overall, during the follow-up period (up to 10/2024), 44% of patients underwent >5PETs, and the median number of PETs performed per patient per year was 2.2. In conclusion, our study identified a substantial overuse of PET in the management of mCRC in Israel, marking a divergence from clinical guidelines. More research is needed to assess this in other diseases and in other countries/regions.

## Introduction

In all healthcare systems around the world, particularly those that are resource constrained, the efficient use of resources is essential for maximizing health outcomes. This relates to both therapeutic and diagnostic modalities. In oncology, imaging options include either standard computed tomography imaging (CT) or positron emission tomography (PET). Although PET adds additional information, it has been deemed unnecessary in some situations, and its cost is usually higher. For example, in Israel the cost of a PET is approximately 5 times the cost of a standard CT.^1^ Nevertheless, overuse of this imaging modality has been documented.^2^

In metastatic colorectal cancer (mCRC), imaging is needed to assess response to systemic therapy such as chemotherapy. Standard CT is considered appropriate for this purpose in most situations, and guidelines clearly state that PET is not routinely indicated but is supported in the setting of patients with equivocal findings on CT or MRI as well as for patients with contraindications to IV contrast. It is also supported in the setting of potentially curable metastatic disease, and in patients considered appropriate for liver-directed therapies.^3^ We hypothesized that, despite such guidelines, PET is overused in mCRC, and evaluated the existence of overuse and its extent, using a large national payer database.

## Patients and methods

### Study design and population

The study was approved by the Institutional Review Board of Clalit Health Services (CHS); approval number, 0140-22-COM1. The study was granted a waiver for obtaining informed consent due to its retrospective design.

This retrospective cohort study included all CHS members (CHS is the largest health maintenance organization in Israel, covering in 2017 nearly 4.5 million members, representing >50% of the Israeli population), aged 18 and over, who are included in the CHS cancer registry with a diagnosis of colorectal cancer (CRC) that was made between January 2017 and December 2022, and received medications (bevacizumab, cetuximab, or panitumumab) that are used only in the metastatic setting during a period extending from 1 month prior to the registered cancer diagnosis to 1 year following the registered diagnosis. This patient registry is maintained in a central database and includes many data components of medical interest. The national CHS dataset has become well recognized for its potential for real-world medical analyses.^4^^,^^5^ Exclusion criteria included an additional cancer diagnosis, aside from CRC, and less than 1 year of follow-up from the time of diagnosis.

The study evaluated the number of PETs performed for every patient until the completion of follow-up in October 2024.

### Data collection and statistical analysis

The following data were collected for all patients: demographic data including age, gender, and socioeconomic status (based on the 2008 Israeli census, using information such as demography, education, employment, housing conditions, and income); date of CRC diagnosis; and number and dates of PET scans between January 1, 2017 and October 31, 2024. All analyses were descriptive and performed using R version 4.1.1.

## Results

The final cohort included 998 patients (Figure S1, see online supplementary material for a color version of this figure). Table 1 presents the characteristics of the patients and the number of PETs performed. The median age of patients in the cohort was 66 years, and 46% were females. The patients were evenly distributed as per year of diagnosis, between 2017 and 2022.

**Table 1.** Patient characteristics and PET usage.

Characteristic	*N* = 998
**Age, median (IQR), years**	66 (56-74)
**Year of diagnosis, *n* (%)**	
** 2017**	160 (16)
** 2018**	163 (16)
** 2019**	189 (19)
** 2020**	173 (17)
** 2021**	132 (13)
** 2022**	181 (18)
**Socioeconomic score,^a^ *n* (%)**	
** 1**	183 (18)
** 2**	451 (45)
** 3**	322 (32)
** Unavailable**	42 (4)
**Sex, *n* (%)**	
** Male**	541 (54)
** Female**	457 (46)
**Years of follow-up, median (IQR)**	2.2 (1.6-3.4)
**Total PETs per patient, median (IQR)**	5.0 (2.0-8.0)
**PETs per patient per year, median (IQR)**	2.2 (1.3-2.9)

Abbreviations: CT, computed tomography; PET, positron emission tomography.

a Socioeconomic score group based on the 2008 Israeli census (3 represents the highest socioeconomic status, 1 represents the lowest).

**Figure 1.**
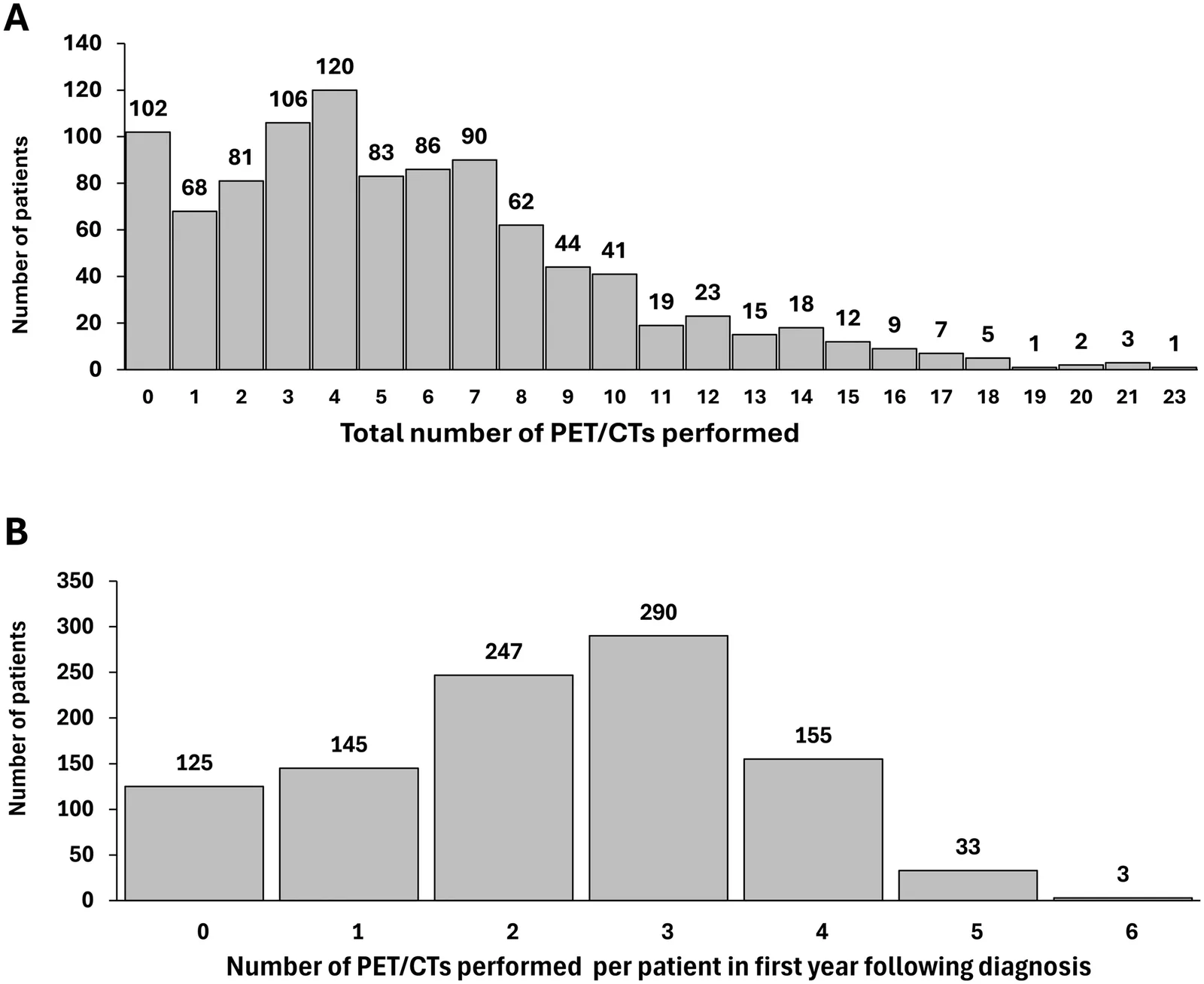
(A) Total number of PETs performed per patient. (B) The number of PETs performed per patient in the first year following the diagnosis.

The median (IQR) follow-up was 2.2 (1.6-3.4) years. The median (IQR) number of PETs per patient per year was 2.2 (1.3-2.9). The number of PETs performed for each patient varied between 0 and 23; the median (IQR) number of PETs per patient was 5.0 (2.0-8.0); 10% did not have any PET and 44% had more than 5 PETs (Figure 1A). In the first year after the CRC diagnosis, 73% of patients underwent more than 1 PET (Figure 1B).

## Discussion

This study suggests a substantial use of PET among patients with mCRC in Israel (medians of 5 PET per patient overall and 2.2 per patient per year). Although there are specific medical reasons that deem PET preferable to standard CT such as severe renal insufficiency, allergy to iodine, and the need to characterize a suspicious lesion, these reasons are relatively rare,^2^ and are unlikely to account for the observed PET use. An additional reason to perform a PET is in the setting of oligometastatic disease when there is potential for cure. Recognizing that such situations account for less than 10% of metastatic cases,^6^ this would translate to a population average of 0.1 PETs per patient. While we have been unable to find robust literature related to the frequency of the need for PET to characterize a suspicious lesion, our clinical practice experience would suggest that this would not account for more than 1 PET on average over the course of a patient’s disease. Taking these two issues into consideration, one could justify a mean of 1.1 PETs per patient. However, in the observed data, the median number of PETs performed was 5. This is almost 5 times what could be considered acceptable.

As part of our policy work, we held various informal conversations with many different clinicians to try to understand more deeply the reasons for this overuse. While there is some private practice cancer care in Israel (predominantly in the form of second opinions), the vast majority of clinical cancer care is performed in the public setting. In this public setting, unusual idiosyncrasies can impact clinical decision-making. For example, in some hospitals, the time taken to receive a radiological report for a standard CT can be vastly longer than that for a PET. Similarly, the waiting time itself for a PET may be shorter. These were cited reasons by some clinicians for preferring PET. Some clinicians also stated that they felt that PET was clinically superior, and were unaware of the stated guidelines.

To resolve this problem of overuse, there is a question of where the control and responsibility should come from—either the payer or the provider. Different approaches may be used to attempt to reduce overuse.^7^ These approaches range on a spectrum from direct control by the payer in its approvals to simply educating physicians. While direct control by the payer in the form of approvals/denials may be effective, it will increase bureaucracy and frustration for clinicians in an already overburdened and bureaucratic healthcare system. Conversely, educating clinicians regarding the appropriate imaging technique is a more gentle approach^8^; however, its effect would be expected to be much more minimal. While these examples represent two extremes, perhaps a more centrist approach may be to employ a more value-based approach, where the financial risk is balanced between payer and provider. One example of how to do this would be for the payer to allow an average of 1-2 PETs per patient journey, but anything beyond that would not be reimbursed, thus creating a financial incentive for the provider to reduce overuse.

In conclusion, our findings highlight the overuse of PET for mCRC in Israel. More research is needed to evaluate if such overuse is evident in other disease indications and in other healthcare systems around the world.

## Supplementary Material

oyag336_Supplementary_Data

## Data Availability

The data underlying this article cannot be shared publicly due to privacy reasons. The data will be shared on reasonable request to the corresponding author.
