# Supplementary material for: Use and overuse of positron emission tomography for patients with metastatic colorectal cancer
Source: Oncologist. 2026 Aug 21;31(9):oyag336. doi: 10.1093/oncolo/oyag336 (PMC13529359; doi:10.1093/oncolo/oyag336)

Supplementary Figure 1. Patient flow chart.

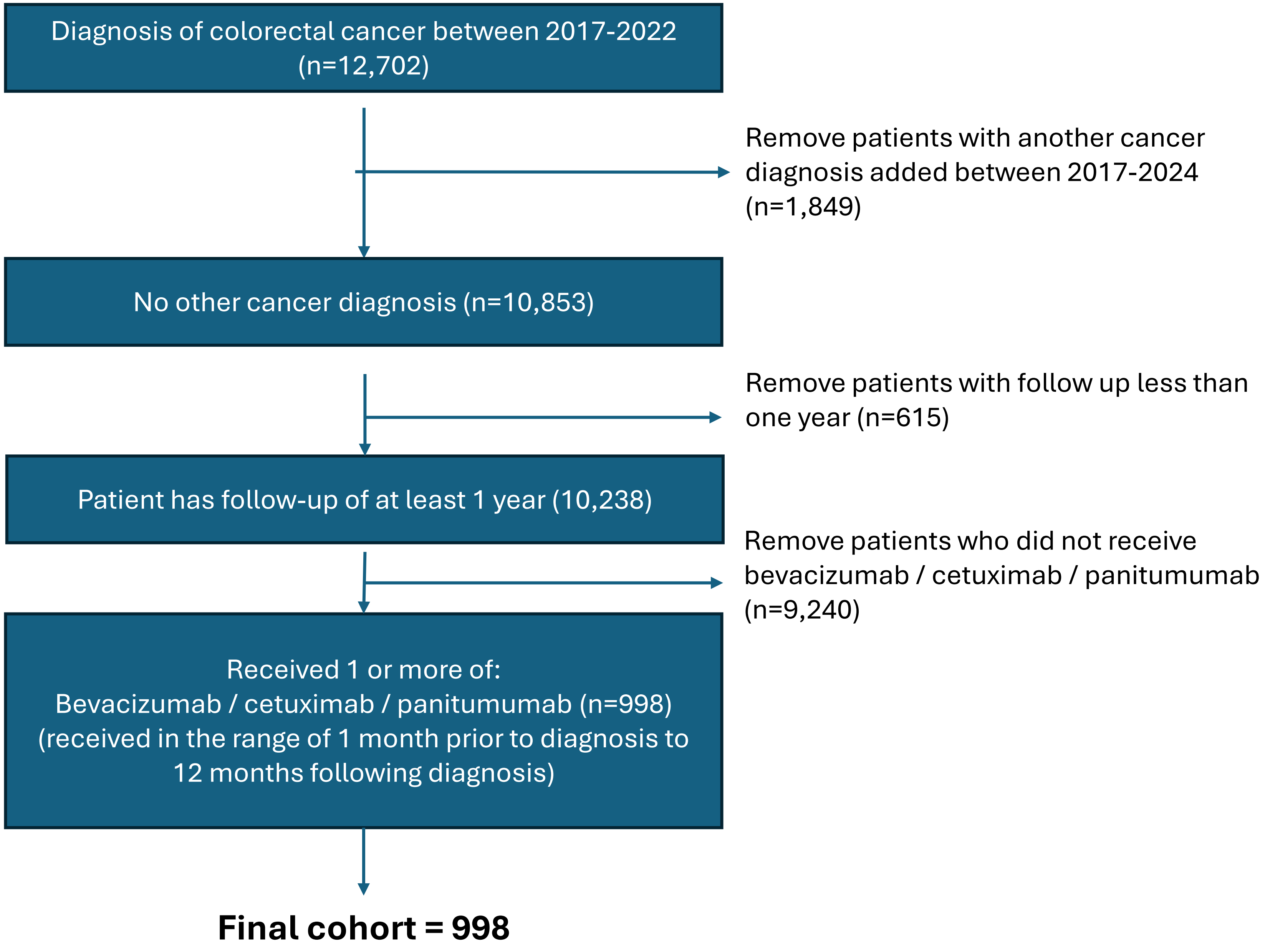

Supplement: Supplementary file 1 [file oyag336_supplementary_data.pdf]
